# Development and testing of a sedation protocol for *Neocaridina davidi*

**DOI:** 10.1038/s41598-024-60158-8

**Published:** 2024-04-25

**Authors:** Diego Rodríguez, Miguel Moscoso, Manuel Desco, Jorge Ripoll, Roberto Fernández

**Affiliations:** 1 Departamento de Bioingeniería, Universidad Carlos III de Madrid, Madrid, Spain; 2 Departamento de Matemáticas, Universidad Carlos III de Madrid, Madrid, Spain; 3 Instituto de Investigación Sanitaria Gregorio Marañón, Madrid, Spain; 4 Centro de Investigación Biomédica en Red Salud Mental (CIBERSAM), Madrid, Spain; 5 Centro Nacional de Investigaciones Cardiovasculares Carlos III (CNIC), Madrid, Spain; 6 Departamento de Física, Ingeniería de Sistemas y Teoría de la Señal, Universidad de Alicante, Alicante, Spain

**Keywords:** Optics and photonics, Animal behaviour, Imaging, Model invertebrates

## Abstract

*Neocaridina davidi*, a small freshwater shrimp native to Asia, specifically China, Japan, Korea, and Vietnam, possesses remarkable resistance to poor water quality and offers various advantages over other invertebrate species to examine crucial issues in neuroscience and other related areas. These advantages include robustness, ease of maintenance, and transparency, making them useful for in vivo studies with optical imaging techniques. Despite its suitability for research purposes, particularly in the fields of imaging and fluorescent techniques, the lack of attention given to this species has resulted in the absence of a robust and replicable sedation protocol for immobilization and safe manipulation. Consequently, researchers face challenges in performing experimental procedures while minimizing harm to this specimen. In this study, we have developed and evaluated a simple sedation protocol specifically designed for *Neocaridina davidi*, assessing its effectiveness using light microscopy and image processing.

## Introduction

Animal testing using vertebrates has been a widespread practice during the past years, for example, as a necessary step in the process of a drug development previous to clinical trials^1^, to study the effects of medical procedures^2^, or in vaccine research^3,4^. However, the robust regulation by law and ethical considerations have strongly limited the use of vertebrate animals in research^2,5^. In addition, animal experimentation with vertebrates presents other important drawbacks, including the need for skilled personnel, time-consuming protocols, and high costs^2,6^. As a result, researchers are increasingly turning their attention to invertebrates as alternative model organisms^7–9^. Besides, invertebrates offer several advantages over vertebrates: they have shorter lifecycles, produce numerous offspring, exhibit relatively simpler anatomical structures, are typically smaller in size, require lower housing expenses, and are less prone to disease transmission within colonies^2,10^.

In fact, the use of invertebrate animals has resulted in significant discoveries across a wide spectrum of biological and medical fields, such as aging processes^11,12^ or the study of processes related to the nervous system of vertebrates^8,13^. The species used for these studies range from terrestrial invertebrates, such as nematodes or insects, to freshwater and marine life, including planarians, crustaceans, mollusks, and many others^7^. Nevertheless, the majority of research conducted on invertebrates has predominantly focused on two species: *Drosophila melanogaster*, commonly known as the fruit fly, and *Caenorhabditis elegans*^7^, a free-living transparent nematode.

In this paper, we study sedative effects of eugenol over the *Neocaridina davidi* shrimp species. We evaluated its sedative capabilities, and developed a first approach for a sedation protocol for imaging applications specifically designed for this small invertebrate. The *Neocaridina davidi* is a small freshwater shrimp (from 2 to 2.5 cm) originating from Asia, specifically from China, Japan, Korea, and Vietnam^14^, that has received very little attention in research as an experimental model. Yet, it has very interesting characteristics that might facilitate new insights about, for example, how nervous systems work.

This particular shrimp exhibits exceptional resilience to suboptimal water conditions, making it highly advantageous compared to other species that are less robust under adverse conditions. It can thrive within a broad range of temperatures (21–27 °C) and pH levels (6–7.5), simplifying the conduct of extensive behavioral studies^15^. Furthermore, beyond its adaptability, this shrimp species demonstrates complex behavior, engaging in various activities such as social interaction, foraging, resting, and reproduction^16,17^. It possesses the ability to recognize and communicate with conspecifics and even other species through olfactory, contact, or visual signals^18,19^. Furthermore, periodic molting serves as an indicator of its well-being and stability^17,20^ . Additionally, certain varieties of *Neocaridina* shrimp are transparent, opening up possibilities for advanced imaging techniques like Light Sheet Microscopy, enabling three-dimensional visualization.

Nevertheless, their inherently restless nature poses challenges in terms of manipulation, as they tend to be difficult to control and frequently exhibit movement during testing. The application of sedation techniques can effectively address these difficulties by allowing researchers to conduct necessary procedures while minimizing resistance and reducing potential harm to the specimens. Since the *Neocaridina davidi* shrimp has received little attention in research, there is currently no sedation study available, similar to what exists for other shrimp species^21–24^. In particular, we have used eugenol as sedative agent. Eugenol is an aromatic phenol that exhibits anesthetic effects, making it an effective sedative for various aquatic species, including fishes and crustaceans. It has been widely used to reduce stress during handling, transportation, and surgical procedures, as well as to induce anesthesia for scientific research in aquaculture settings^21–23,25^. Its effectiveness has been also proved for other shrimp species as *Fenneropenaeus indicus, Litopenaeus vannamei* or *Macrobrachiurn rosenbergii*^21,23,26^. Therefore, the main aim of this study is to understand and characterize the response of *Neocaridina davidi* to sedatives, specifically to eugenol. Thus, we carried out a set of initial experiments.

## Materials and methods

For sedation, we prepared a mixture of eugenol and ethanol. The baseline concentration of eugenol was based on previous studies on crustaceans other than *Neocaridina davidi*. For example, Vartak et al.^27^ used a concentration of 75 mg/L for *Macrobrachium rosenbergii*, with a specimen size of 13.42 mm, Parodi et al.^28^, used 175 mg/L for *Litopenaeus vannamei*, a species with a size of 5 mm, Li et al.^29^ used a concentration of 200 mg/L for *Palaemonetes sinensis* for 25 mm species size and Palomera et al.^30^ used 300 mg/L for *Macrobrachium tenellum*, with a size of 46 mm. Table 1 shows the data adjusted for specimen size by dividing the concentration of sedative used by the average size of the specimen. Concentration of sedative, C, is expressed as mg/L considering a density of eugenol of 1 g/mL.

**Table 1:** Eugenol concentrations in previous studies on crustaceans.

Species	Size (mm)	Eugenol concentration (mg/L)	Adjusted concentrations C/mm
*M. rosenbergii*	13.42	75	5.59
*L. vannamei*	5	175	35
*P. sinensis*	25	200	8
*M. tenellum*	46	300	6.52

The table shows species names, sizes, applied eugenol concentrations, and adjusted concentrations (C/mm), calculated by dividing eugenol concentration by average specimen size. Concentration unit (C) is in mg/L.

### Sedative effects of eugenol over *Neocaridina davidi*

For a first evaluation of the effects of eugenol, and based on mentioned studies, we considered a conservative adjusted sedation of approximately 5 C/mm for the smallest *Neocaridina davidi* size, 20 mm, leading us to set the safest baseline at 100 mg/L. The mentioned studies also recommend the use of ethanol at a volume ratio between 5:1 and 10:1 with respect to eugenol to facilitate the dissolution of eugenol in the sedative bath. Although no adverse effects were reported with the use of ethanol^27^, for safety reasons, a 5:1 ratio was used. The volume of sedative required to achieve a desired concentration was calculated as

1 $$\begin{aligned} V_s = \frac{C_s \cdot V_B}{\rho _s}, \end{aligned}$$

where $V_s$ represents the volume of sedative, $C_s$ the concentration of sedative, $V_B$ the total volume of the bath and $\rho _s$ the density of the sedative. This equation was also used to determine the concentration from a known amount of sedative.

**Figure 1:**
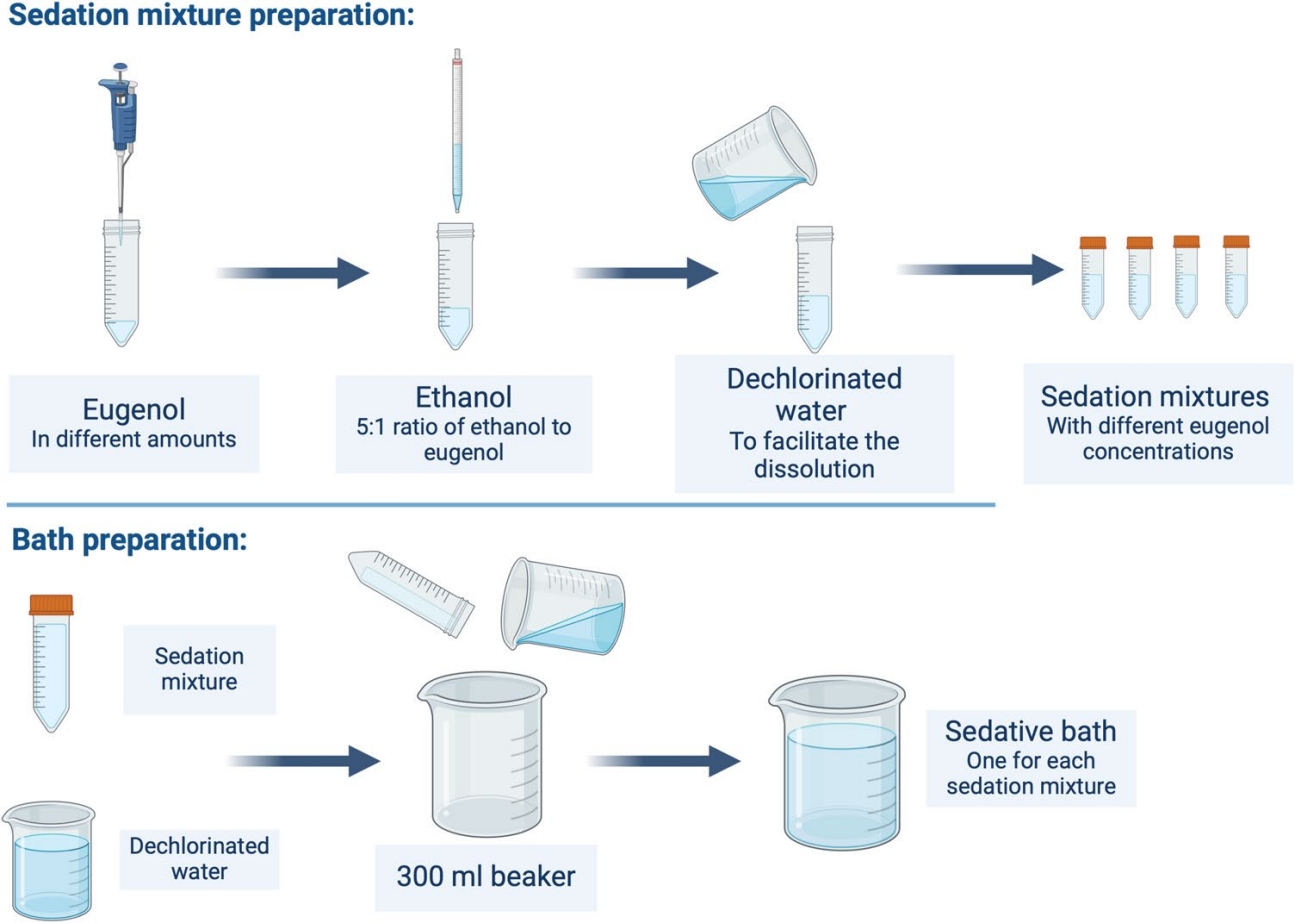
Sedation mixture and bath preparation schemes. Baths with different concentrations of the sedative were prepared following this scheme to assess sedative effects of eugenol over *Neocaridina davidi*.

Figure 1 shows the sedation mixture preparation in 50 mL Falcon tubes. In some of the preparations, a small amount of dechlorinated water was added to facilitate the dissolution of the sedative. Thus, each Falcon tube contained the specified amount of sedative, its corresponding 5:1 volume of ethanol, and, when necessary, a small quantity of water. Once prepared, the sedation mixture was poured and mixed into the containing vessel. Then, dechlorinated water was added until the total volume was reached (Fig. 1, lower panel). For all experiments, the total volume of the bath was 300 mL and the vessel containing the specimens during sedation was a standard 300 mL beaker.

Eight different baths were prepared to reach the lowest feasible level of *real* and *pragmatic* sedation needed for the subsequent procedures. *Real* sedation denotes the stage at which the specimen’s regular functions decelerate or halt, while *pragmatic* sedation refers to the point where the specimen can be handled to obtain the necessary samples without disruptions. In both cases, the specimen must be capable of a complete recovery once removed from the sedative bath to consider the sedation as successful.

**Figure 2:**
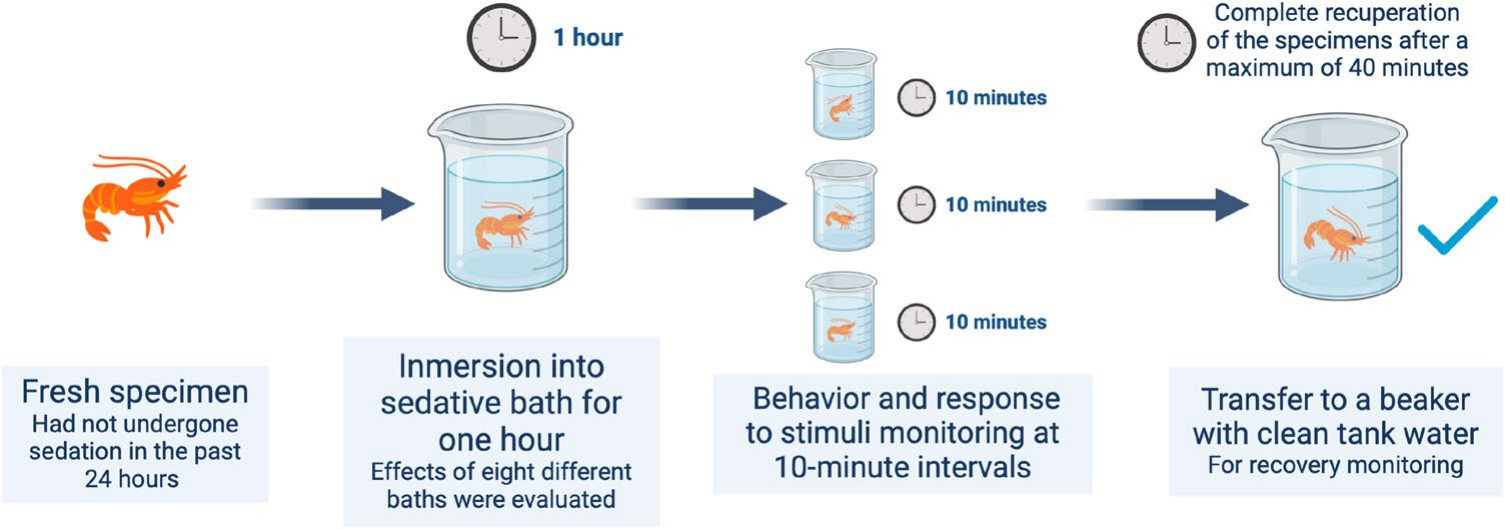
Scheme outlining the procedure for evaluating the sedative effects of eugenol. This process was employed for the preparation of each of the eight different baths under evaluation.

Figure 2 shows the sedation procedure. It consisted in immersing a fresh specimen, that had not undergone sedation in the past 24 h, into the sedative bath for one hour. The behavior and response to stimuli was monitored at regular 10-min intervals during the immersion period. The primary objective of these initial experiments was to evaluate the sedative properties of eugenol. We considered that a preparation was successful when a *real* sedation state was achieved. However, for more complex procedures, a *pragmatic* sedation approach may become necessary. Thus, we developed a basic sedation protocol to address this need.

### Development of a sedation protocol

**Figure 3:**
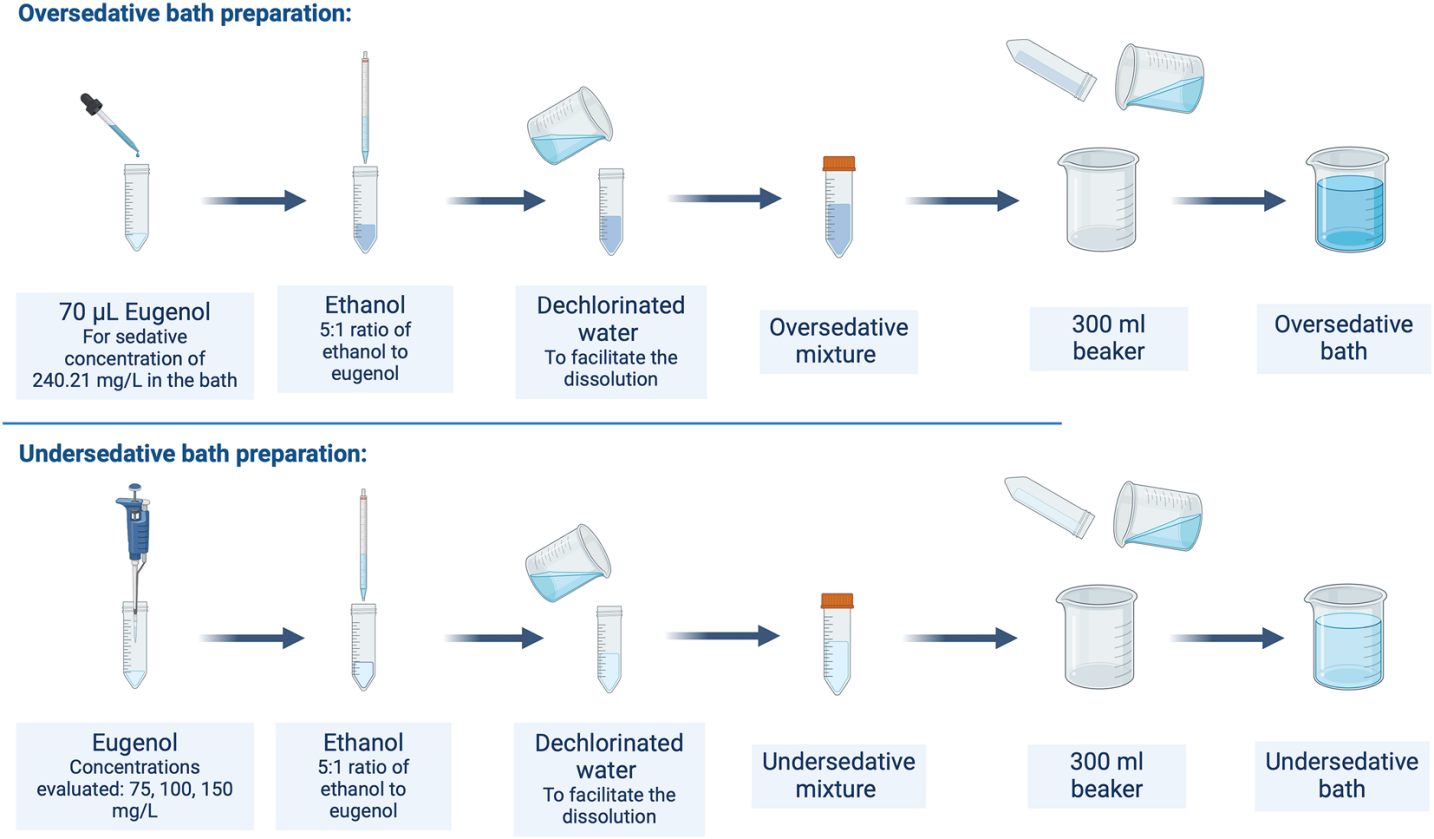
Oversedative and undersedative baths preparation schemes. For the undersedative bath, three different baths were prepared with eugenol concentrations of 75, 100, and 150 mg/L.

After evaluating the effects of eugenol on the specimens, we carried out a new set of experiments to establish a basic sedation protocol for image acquisition procedures ensuring continuous *pragmatic* sedation. Based on the experiments detailed in the previous section, we prepared (i) oversedative bath and (ii) undersedative baths as it is illustrated in Fig. 3. These baths were designed to reduce sedation time and prolong the sedative effects while using lower concentrations of sedatives. The oversedative bath was specifically formulated to induce rapid sedation, while the undersedative bath was prepared to maintain sedation after transferring the specimen from the oversedative bath.

In the preparation of oversedative bath, bottle droppers (Glassco) served as a model for volume measurement with an approximate dispensing rate of 50 μL per drop. However, due to the low precision of droppers, we consider the upper limit of 70 μL per drop to prepare the sedative baths^31^. Thus, the oversedative bath contained a drop of 70 μL of eugenol, representing the highest volume achievable with a single drop from the dropper in the specified range. Following the steps shown in Fig. 3, a 300 mL bath was set up adding 5:1 ratio of ethanol to eugenol in volume. The density of the eugenol used was determined in the laboratory to be 1.0295 g/mL. Using Eq. (1), the final concentration of sedative in the oversedative bath was 240.21 mg/L. This is similar to typical eugenol concentration used for research on similar-sized crustaceans^24^. It is important to highlight that prolonged exposure to oversedative baths for more than 20 min might poses significant risks to the specimens and might cause death.

On the other hand, the undersedative bath used for the sedation protocol had an eugenol concentration of 100 mg/L and 5:1 ratio of ethanol to eugenol in volume, which proved to be effective in maintaining specimen sedation. We also evaluated undersedative baths with eugenol concentrations of 75 mg/L and 150 mg/L (Fig. 3, lower panel).

**Figure 4:**
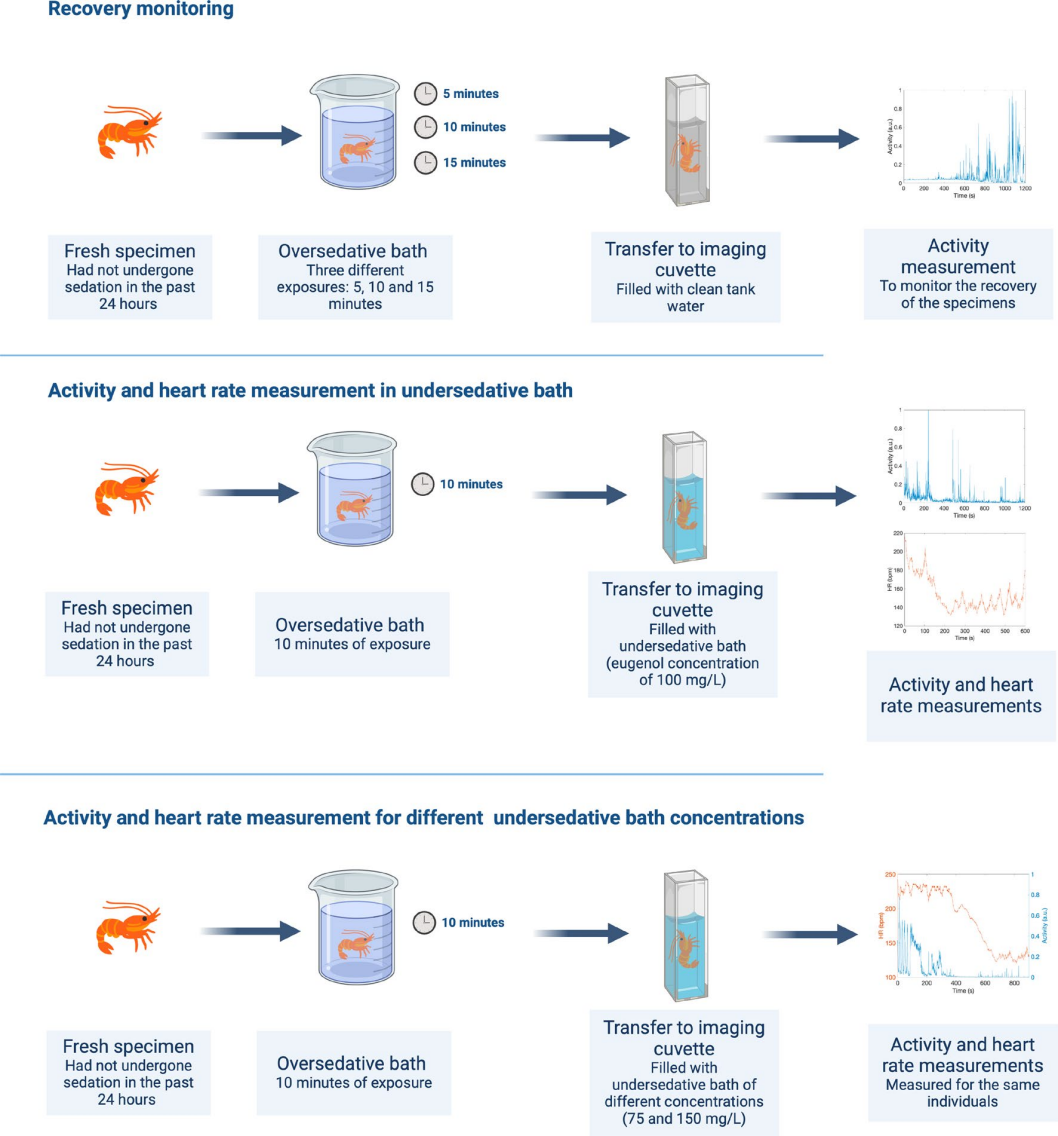
Schematic representation of the experiments carried out to assess the sedation protocol: First, activity measurement in clean tank water after an oversedative bath to determine recovery time. Second, activity and HR measurement in undersedative bath to study the persistence of the intended sedative state. Third, evaluation of the effects of varying eugenol concentrations in the undersedative bath.

Figure 4 shows a schematic representation of the different experiments carried out to assess the sedation protocol. For this series of experiments, fresh specimens were exposed to oversedative baths of 5, 10, and 15 min. After the oversedative bath, the specimens were placed in a cuvette and imaged through light microscopy at 100 ms intervals. Recovery from sedation was monitored by measuring the movement, or activity, in clean tank water after these three different exposure times to the oversedative bath (5, 10 and 15 min). On the other hand, to investigate the sedative effects of undersedative baths, the activity and HR were measured under undersedative baths of different sedative concentrations (75, 100 and 150 mg/L) after 10 min of an oversedative bath. This ensured sufficient sedation while avoiding prolonged exposures to high concentrations of eugenol. As depicted in Fig. 4, during experiments with the 100 mg/L undersedative bath, activity and HR were measured separately for different individuals. However, in the experiments assessed to evaluate the effects of 75 mg/L and 150 mg/L undersedative baths, both parameters were simultaneously measured for each specimen.

**Figure 5:**
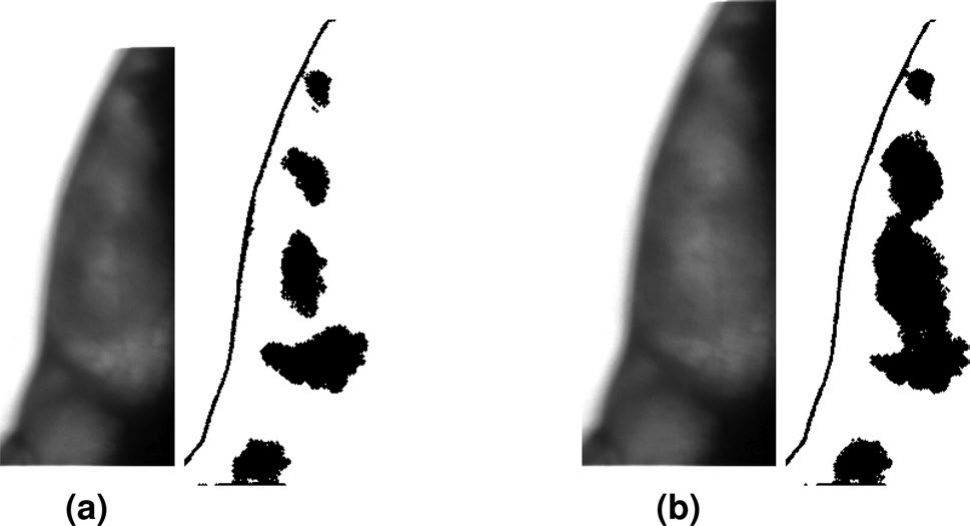
ImageJ visualization of Contracted (**a**) and dilated (**b**) shrimp’s heart threshold for analysis of the hearth rate.

To assess the movement, or activity, of the specimen, we created a macro in *ImageJ*^32^. This macro was used to calculate the difference between two time-subsequent images using the *Image Calculator* functionality, and then measuring the average pixel value of the difference image. The HR of the shrimp under sedation was analyzed by processing the area surrounding its heart. As the heart dilated and contracted, the variations in brightness of the area were analyzed. A threshold allowed us to isolate this pattern as cyclically pulsating dark areas that were correlated to the shrimp’s heartbeat (Fig. 5). However, it is important to note that this method is sensitive to noise, as random fluctuations in pixel values could lead to local maxima that do not align with actual dilations. To mitigate false positives, we applied grayscale morphology by eroding and then dilating the images. Then, a custom-made script developed in Matlab R2023b was used to find local maxima by determining the number of frames between these local maxima and to convert these values into a HR measurement in beats per minute.

## Results and discussion

### Sedative effects of eugenol over *Neocaridina davidi*

Our initial approach involved assessing the sedative effects of eugenol on *Neocaridina davidi*. This was done by preparing different sedative baths below the mentioned safest baseline of 100 mg/L. A total of eight baths, with different concentrations of eugenol from 25 to 80 mg/L, were prepared. The sedative effects of each bath were evaluated for a total of 24 individuals, distributed evenly among the three replicates conducted for each experiment, by measuring the required time to induce *real* sedation. The respective times for each individual and the concentrations used are summarized in Table 2. We emphasize that the primary objective of this study was to evaluate the sedative properties of eugenol, focusing on its effectiveness, rather than on the time required to induce sedation in each case. The cases in which no sedation was induced after one hour of sedative bath are indicated in the table as “NS”. Nevertheless, we note that, in the majority of the cases, the specimens were sedated after 30–40 min of exposure to the sedative bath, in concordance with the sedation times exhibited by other shrimp species^28,29^ under comparable eugenol concentrations. On the other hand, these experiments revealed individual-specific responses to sedation and, in some instances, resistance to the effects of low concentrations of sedative.

**Table 2:** Sedation induction times for various eugenol concentrations with a total of 24 individuals.

Series eugenol concentration (mg/L)	Series sedation induction time (min)		
	Series replicate 1	Series replicate 2	Series replicate 3
25	NS	40	40
30	NS	20	NS
40	NS	30	50
50	10	40	NS
60	NS	20	30
70	30	30	50
75	10	NS	20
80	30	20	40

Eight baths, ranging from 25 to 80 mg/L of eugenol, were assessed. The experiment was replicated three times, with three individuals observed per bath.

After sedative baths, the evaluated specimens were transferred to a beaker containing clean tank water to monitor their recovery. All specimens exhibited complete recuperation without observable signs of harm after a maximum of 40 min submerged into the clean tank water. Oversedation was also evaluated with a sedative bath preparation containing 70 μL of eugenol into a 300 mL bath. As stated above, this resulted in a eugenol concentration of approximately 240.21 mg/L. In this case, 10 min of immersion in this preparation consistently induced *real* sedation in the 24 evaluated shrimps, with an average duration of the sedation of 60 min. Thus, confirming eugenol’s sedative efficacy over *Neocaridina davidi*.

### Development of a sedation protocol

**Figure 6:**
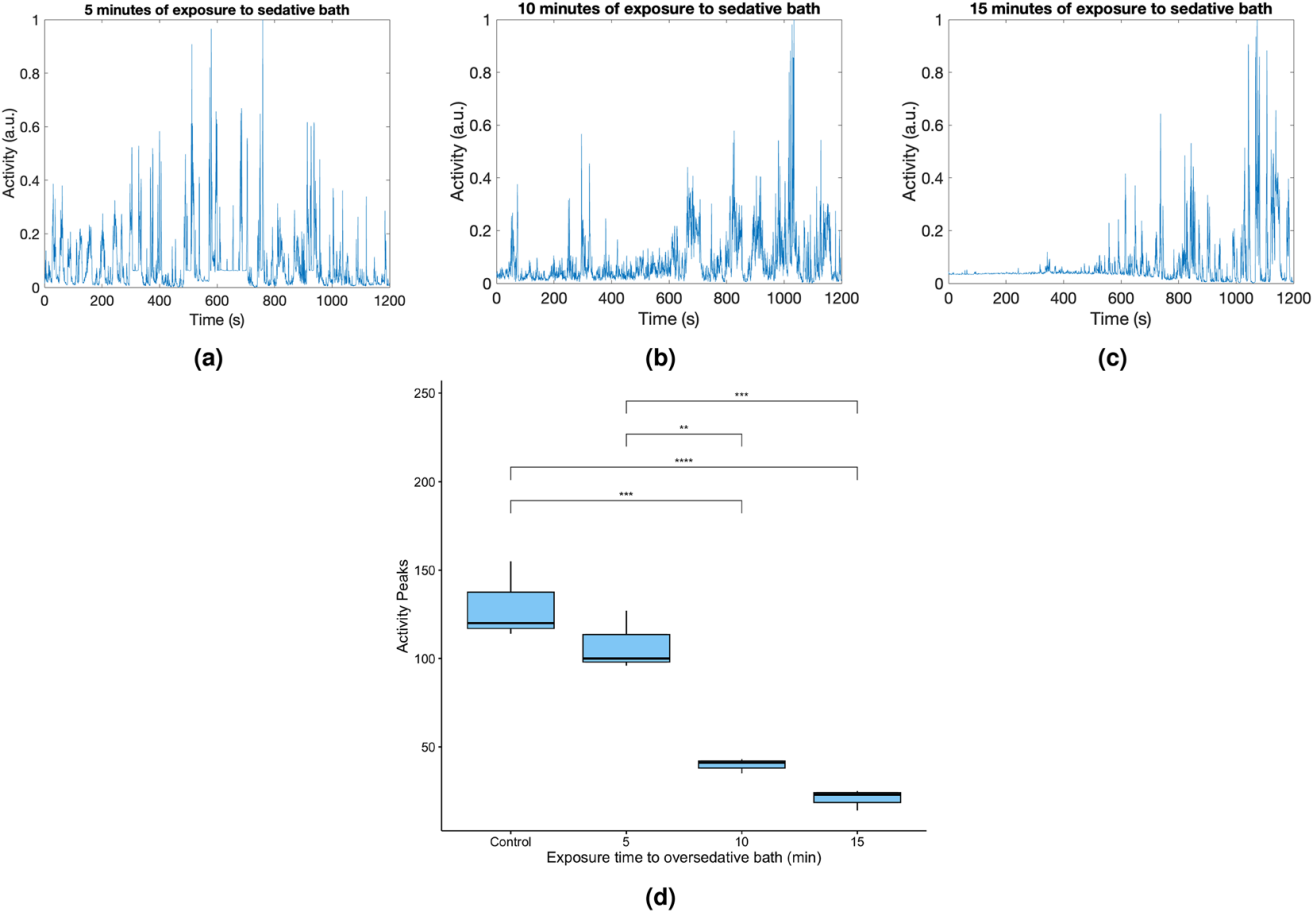
Activity plot of three representative specimens during 1200 s after exposure to the oversedative bath for 5 (**a**), 10 (**b**), and 15 (**c**) min. Activity peaks were measured during the first 10 min after these three different exposures to the oversedative bath (**d**). For comparison purposes, (d) includes the distribution of activity peaks for control individuals not exposed to the oversedative bath.

Once sedative effects of eugenol were assessed, we evaluated the proposed sedation protocol. First, we monitored the recovery of 9 fresh specimens by measuring their activity in clean tank water after 5, 10 and 15 min of exposure to the oversedative bath (Fig. 6a–c, respectively). After 10 and 15 min of exposure to the oversedative bath, the activity levels, measured as activity peaks, were observed to be low until approximately 10 min of activity monitoring in the undersedative bath, clearly indicating the sedative effects of eugenol. As expected, the sedative effect was more pronounced after 15 min of exposure to the oversedative bath. The specimens started to recover 10 min after submersion in the clean tank water, completing an observable full recovery within 60 min in the clean tank water. This recovery time aligns with that reported for similarly sized specimens of other shrimp species treated with eugenol, with recovery times ranging from 17 to 60 min^27,29,30^. In Fig. 6a, we observe that the specimen was not fully sedated after 5 min of exposure to the oversedative bath, observing activity during the whole duration of the experiment. Figure 6d shows the distribution of activity peaks measured during 10 min after the three different exposures to the oversedative bath (5, 10 and 15 min). One-way Analysis of Variance (ANOVA) was used to confirm statistical differences between exposure times, followed by a post hoc Tukey test. The results of the analysis revealed a statistically significant difference between the groups (F(3, 8) = 39.96, *p* < 0.0001), indicating variations in activity levels following exposure to the oversedative bath. Post hoc Tukey test indicated that shrimp exposed to a 5-min sedative bath exhibited activity levels not significantly different from the control group, meaning that this bath did not produce complete sedation. On the other hand, individuals exposed to 10- and 15-min oversedative baths showed reduction of the activity.

For complete sedation, exposures between 10 and 15 min were required. However, in order to avoid significant risks to the specimen, it is important to not extend the exposure to the oversedative bath beyond 20 min^29,33^. Based on these results, we set an oversedative bath exposure time of 10 min, avoiding prolonged exposure to eugenol concentrations above 100 mg/L.

**Figure 7:**
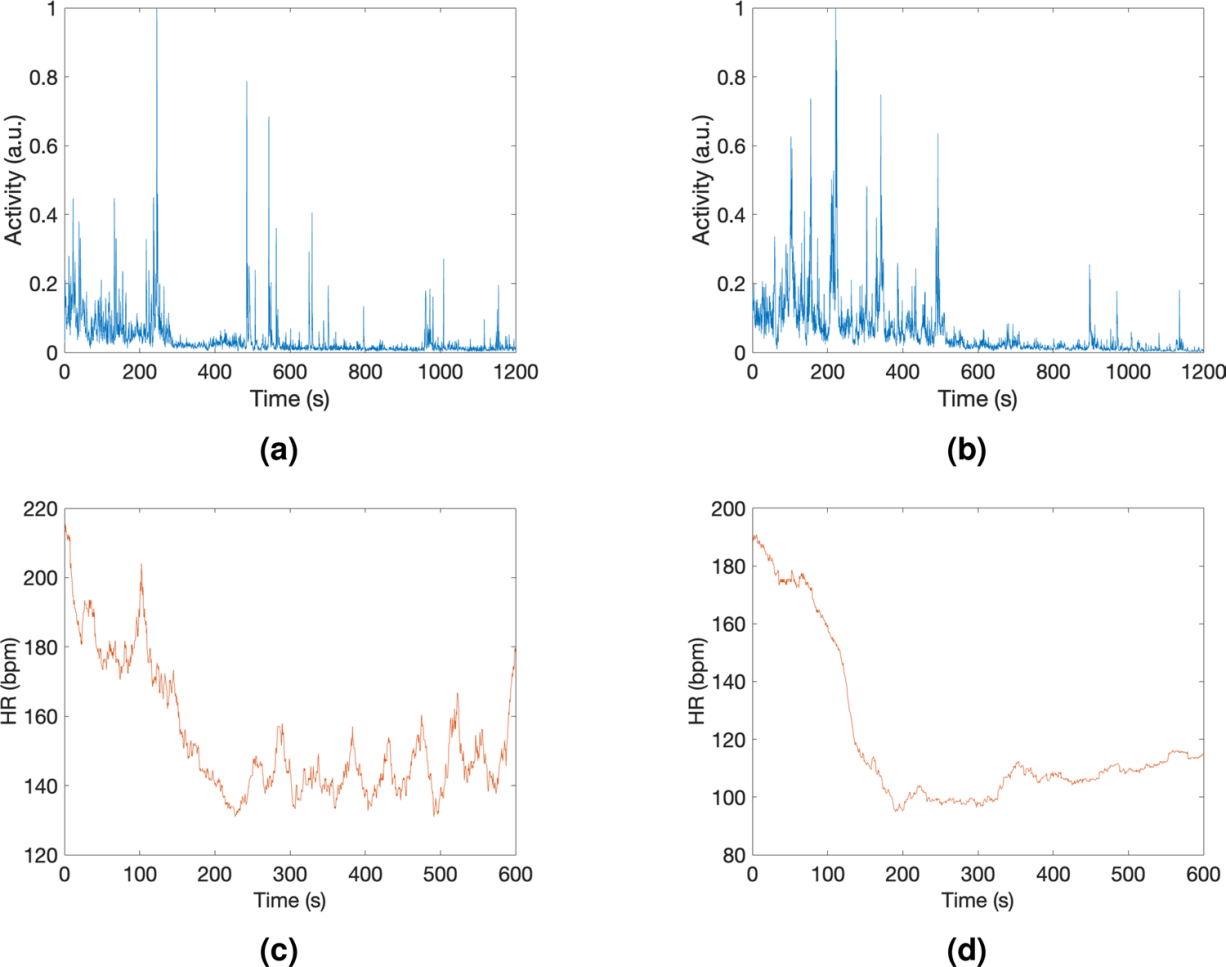
Activity plot ((**a**) and (**b**)) and HR measurement ((**c**) and (**d**)) of four different specimens during undersedation bath with a sedative concentration of 100 mg/L after 10 min of exposure to the oversedative bath.

For measuring activity and HR, 10 fresh specimens were monitored in an undersedative bath after an oversedative bath. Figure 7 show these measurements for four different individuals. The activity measurements indicated an initial period of stabilization for all the individuals after immersion into an undersedative bath of around 5 min (272 ± 24 s) until the mean value of the activity dropped below the 5%. Nevertheless, the overall low measured activity (mean value of activity below 5%) confirmed that undersedation bath effectively keeps *Neocaridina davidi* under a *pragmatic* sedation state using a lower concentration of sedative compared to the oversedative bath or the concentrations typically employed for *pragmatic* sedation in similarly sized individuals of other species^29,34^, which have been associated with elevated mortality rates in small-sized specimens or post-larval individuals^27,28^. Therefore, this protocol allows imaging of *Neocaridina davidi* for extended durations while ensuring continued sedation and safeguarding its well-being throughout the process.

Regarding the HR measurements, we observed a noticeable drop in HR after a mean of 260 ± 38 s after immersion into a 100 mg/L eugenol concentration undersedative bath coming from 10 min immersion in an oversedative bath. Figure 7c,d show a reduction from 218 to 135 bpm and from 190 bpm to 95 bpm, respectively, for two individuals. In all the cases analyzed in this study, the specimens exhibited elevated initial HRs upon transfer from the oversedative bath to the undersedative bath. This elevated baseline may be attributed to the stress induced during the transfer process, requiring a period of stabilization before the effects of the undersedative bath become apparent. Still, all the cases were sedated after less than 5 min of immersion into the undersedative bath. In the absence of the first oversedative bath, as previously demonstrated, this concentration of eugenol would typically necessitated longer exposure times to induce sedation in the specimens.

**Figure 8:**
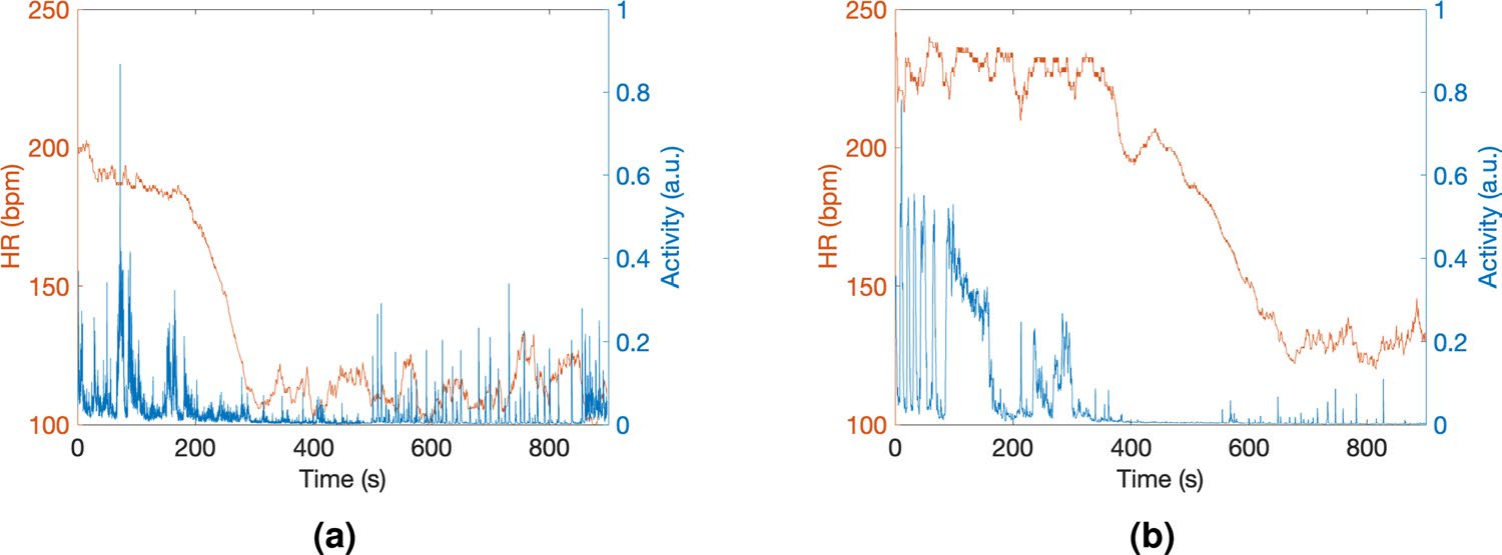
HR and Activity Levels of two individuals of *Neocaridina davidi* under undersedative baths with eugenol concentrations of 75 mg/L (**a**) and 150 mg/L (**b**) showing the individual-specific response to sedation.

Finally, to gain a better understanding of the effects of eugenol on *Neocaridina davidi*, we simultaneously measured the HR and activity levels for each individual when immersed into 75 mg/L and 150 mg/L eugenol concentration undersedative baths. Following the methodology of previous experiments, 10 fresh specimens were initially exposed to an oversedative bath for 10 min before being transferred to the undersedative bath. Five individuals were exposed to the 75 mg/L eugenol concentration bath, and the remaining five to the 150 mg/L eugenol concentration bath. In all cases, exposure to the undersedative bath for 10 min led to a reduction in HR to values between 100 and 150 bpm (Fig. 8). Activity levels were also diminished, indicating that the lowest activity levels corresponded to the point when the HR began to decrease. These results confirm that eugenol not only reduces the activity of the specimens but also induces a decrease in HR, as was observed in previous experiments. Although we observed shorter mean time for activity decrease for 150 mg/L bath, sedative effects for concentrations below the oversedative range (240.21 mg/L) exhibited greater dependence on individual variability rather than the specific eugenol concentration used, as we showed in Table 2. Therefore, using a higher eugenol concentration does not necessarily imply an increase in sedation effects for all the cases, such as the time needed to achieve a stable low HR after transferring from the oversedative bath. Furthermore, no statistically significant difference was observed through ANOVA for the different undersedative concentrations studied: F(2, 12) = 3.18, *p* = 0.07 for activity and F(2, 12) = 0.314, *p* = 0.736 for HR.

**Figure 9:**
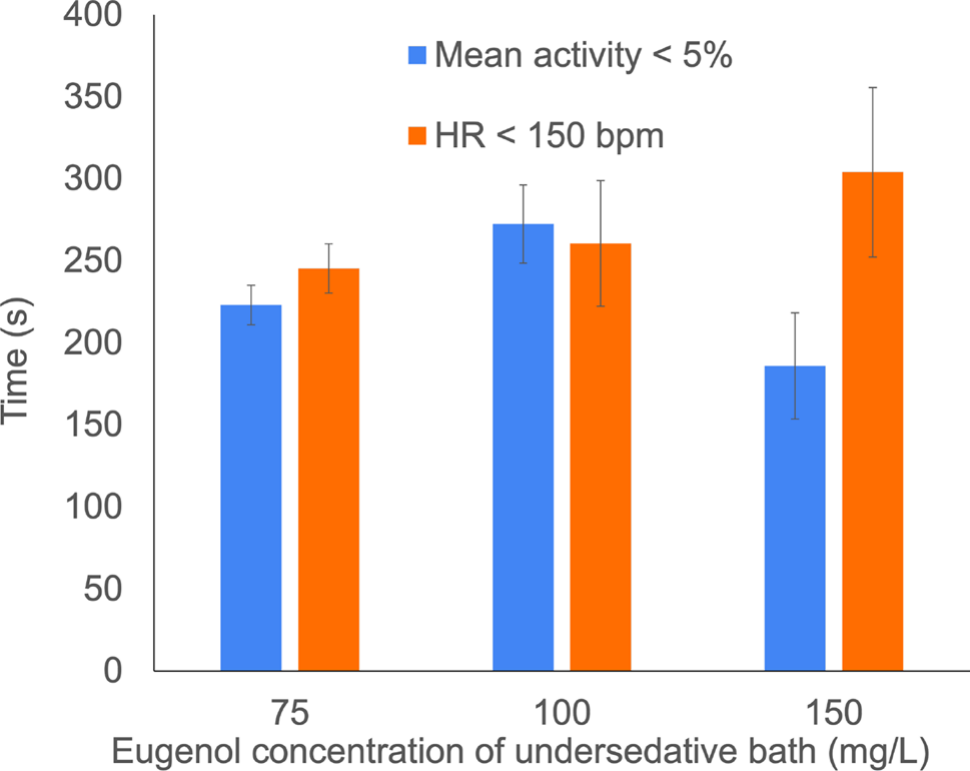
Mean exposure time required to reduce the mean activity below the 5% (blue) and the HR below 150 bpm (orange) during undersedative bath of 75 mg/L, 100 mg/L and 150 mg/L eugenol concentration after 10 min of exposure to oversedative bath.

For the undersedative bath of 75 mg/L eugenol concentration (Figs. 8a and 9), the mean exposure time required to reduce the mean activity below the 5% and the HR below 150 bpm were 223 ± 16 s and 245 ± 19 s, respectively. These values were similar to the ones measured during undersedative bath of 100 mg/L eugenol concentration (272 ± 24 s and 260 ± 38 s, respectively) . Notably, one of the specimens showed higher resistance to the 150 mg/L undersedative bath, as it is shown in Fig. 8b. In this case, the HR began to decrease after 400 s of exposure to the bath, reducing its HR below 150 bpm after 594 s. However, the low activity persisted after transferring from the oversedative bath and further decreased after 3–6 min of exposure to the undersedative bath. The mean exposure time required to reduce the mean activity below the 5% and to reduce the HR below 150 bpm in the 150 mg/L eugenol concentration undersedative bath were 186 ± 32 s and 304 ± 51 s, respectively (Fig. 9).

## Conclusions

In this work, we have studied sedative effects of eugenol over the *Neocaridina davidi* shrimp species. First, we evaluated its sedative capabilities, immersing the individuals in baths with different concentrations of eugenol. The results confirmed eugenol’s sedative properties, with most individuals being sedated within one hour of exposure to the eugenol bath. Notably, for eugenol concentrations below those used for oversedative baths, we observed individual-specific responses to sedation, and, in some cases, resistance to undersedative concentrations. On the other hand, oversedative concentrations of 240.21 mg/L consistently induced sedation for 60 min after 10 min of immersion. In the case of the undersedative bath, all specimens exhibited complete recuperation without observable signs of harm after a maximum of 40 min submerged into clean tank water.

We also developed a first approach for a sedation protocol for imaging applications with *Neocaridina davidi*. This sedation protocol involves a 10-min oversedative bath followed by an undersedative bath to maintain a secure sedation. This procedure reduced the overall sedation time and the risks caused by an excessive exposure to eugenol, while prolonging the sedative effect. The protocol’s effectiveness was confirmed by measuring the activity and HR of different individuals. The undersedative bath successfully maintained low activity levels, which were further reduced after 3–6 min of exposure. Additionally, after stabilizing from the transfer out of the oversedative bath, the HR decreased and remained at an average of 150 bpm after 3–10 min of exposure to the undersedative bath.

This study is a first step in developing sedation protocols for *Neocaridina davidi*. It shows the potential of eugenol-based sedation protocols for practical applications in *Neocaridina davidi* imaging and handling. Further studies can explore individual responses to better understand the properties of eugenol and its effects on this invertebrate. Additionally, investigating other sedative agents, like essential oils from Lippia alba or Aloysia triphylla, could be an interesting direction for future developments in this area.

## Data Availability

The datasets used and/or analysed during the current study available from the corresponding author on reasonable request.
